# Management of anticoagulated patients in dentoalveolar surgery: a retrospective study comparing bridging with heparin versus unpaused vitamin K antagonist medication

**DOI:** 10.1186/s12903-021-01464-9

**Published:** 2021-03-05

**Authors:** Mayte Buchbender, Felix Rößler, Marco R. Kesting, Gesche Frohwitter, Werner Adler, Andrea Rau

**Affiliations:** 1 Department of Oral and Maxillofacial Surgery, Friedrich-Alexander-University Erlangen-Nuremberg, Glückstrasse 11, 91054 Erlangen, Germany; 2 Department of Medical Informatics, Biometry and Epidemiology, Friedrich-Alexander-University Erlangen-Nuremberg, Erlangen, Germany

**Keywords:** Anticoagulation therapy, Bleeding, Bridging with heparin, Vitamin K antagonist

## Abstract

**Background:**

The aim of this study was to investigate the occurrence of postoperative bleeding following dentoalveolar surgery in patients with either continued vitamin K antagonist medication or perioperative bridging using heparin.

**Methods:**

A retrospective study was performed analyzing patients who underwent tooth extraction between 2012 and 2017. Patients were retrospectively allocated into two comparative groups: un-paused vitamin K antagonist medication versus bridging using heparin. A healthy, non-anticoagulated cohort with equivalent surgery served as a control group. Main outcome measures were: the occurrence and frequency of postoperative bleeding, the number of removed teeth, the surgical technique of tooth removal (extraction/osteotomy/combined extraction and osteotomy) and the prothrombin time.

**Results:**

In total, 475 patients were included in the study with 170 patients in the group of un-paused vitamin K antagonist medication VG, 135 patients in the Bridging group BG and 170 patients in the control group CG. Postoperative bleeding was significant: CG versus VG *p* = 0.004; CG versus BG *p* < 0.001, BG versus VG *p* < 0.001. A significant correlation of number of the extracted teeth in the BG (*p* = 0.014) and no significance in VG (*p* = 0.298) and CG (*p* = 0.210) and in the BG versus VG and CG with *p* < 0.001 in terms of surgical intervention extraction. No difference observed in terms of prothrombin time.

**Conclusions:**

Bridging with heparin increases the risk for bleeding compared to un-paused vitamin K antagonist medication. The perioperative management of anticoagulated patients requires a well-coordinated interdisciplinary teamwork to minimize or at best avoid both: postoperative bleeding and thromboembolic incidences.

## Background

Due to the increase in life expectancy and the rising number of patients with cardiovascular diseases, the number of anticoagulated patients continues to increase worldwide [1]. Although the therapeutic anticoagulation management of the underlying diseases (e.g., apoplexy, atrial fibrillation, coronary heart disease, peripheral arterial occlusive disease, heart valve replacement or thrombosis) is primarily an internal medicine issue [2], blood-thinning medication plays also a major role in oral surgery. Dentoalveolar surgical procedures such as tooth extractions, tooth osteotomies or root tip resections are part of the everyday dental practice. For anticoagulated patients, they require a close interdisciplinary coordination between cardiologists, general practitioners and oral surgeons [3]. Inconsiderate perioperative disruption of the anticoagulative medication bears the risk of potentially lethal thromboembolic events for the patients. In their review, Wahl et al. reported 22 embolic events after discontinuation or reduction of anticoagulation, 6 of which ended fatally, whereas no fatal consequences were observed after postoperative bleeding with existing anticoagulation [4]. Although fortunately not being life-threatening in the vast majority of cases, postoperative bleeding following oral surgery causes severe discomfort for the patients, who might be hospitalized and may face follow-up operations for hemostasis.

Anticoagulation therapy can be carried out using various classes of drugs (e.g. vitamin K antagonists, heparin, direct oral anticoagulants), which all intervene differently in the coagulation cascade and have their assets and drawbacks. Despite the availability of substances, which are easier to handle (e.g. direct oral anticoagulants), the coumarin derivative and vitamin K antagonist Phenprocomoun (Marcumar®), is still widely used for the prevention of thromboembolic events in atrial fibrillation or following heart valve replacement or pulmonary embolism. There are widely discussed approaches to the management of vitamin K antagonists prior to oral surgery: suspension for several days [2, 5], temporary bridging with heparin [6, 7], reducing the dosage without bridging [8, 9] or unchanged dosages and hemostasis by local hemostatic measures [2, 5, 10, 11]. Heparin offers the advantage of a good controllability due to its short half-life [12]. However, it is not recommended to interrupt the heparin therapy for surgical interventions [13]. In any case, preoperative coagulation lab testing is essential to evaluate the patients’ level of anticoagulation [14]. When investigating bleeding risks in a cohort of 1884 patients who received a surgical intervention with an adjusted INR of > 2.0 and were either bridged with low-molecular-weight heparin or a placebo, Douketis et al. found the risk of bleeding was 1.3% in the placebo group and 3.2% in the experimental group [15].

In general, available literature on this topic is heterogeneous. Whilst there is a relative consensus pro Bridging regarding major surgery, especially for small-to-medium-sized surgical interventions including oral surgery the recommendations differ considerably even in official guidelines [16, 17]. It was therefore the aim of this study to analyze bleeding complications in a cohort of anticoagulated patients having oral surgery. A special focus was laid on the comparison of Bridging versus un-paused vitamin K antagonist medication.

## Methods

A monocentric retrospective patient cohort analysis was performed in a German university dental clinic, including all patients with a permanent vitamin K antagonist medication, who had oral surgery done between 2012 and 2017 in the clinic or were transferred to the clinic for treatment following oral surgery. As a first step, patient search was conducted by screening the digital clinic documentation system (MCC®, Meierhofer AG, Munich, Germany) and the digital patient file (Soarian Clinicals®, Cerner Health Services, Erlangen, Germany) using the following key words: tooth extraktion, tooth osteotomy, surgical intervention, tooth, bleeding event, Marcumar®, Bridging, heparin, anticoagulation and thromboembolic event. As a next step, further selection of patients was carried out, by including only patients, who had an oral surgical intervention (tooth extraction, tooth extraction and osteotomy or osteotomies). Both, in- and outpatients were considered. Furthermore, all patients with hemorrhagic diatheses or blood-thinning medication other than vitamin K antagonists (e.g. direct oral anticoagulants or platelet aggregation inhibitors) were excluded.

Depending on whether the vitamin K antagonist medication was temporarily paused and substituted by heparin perioperatively (= Bridging) or continued without interruption, we retrospectively allocated the selected patients into two groups: a Bridging group named BG and a vitamin K antagonist group named VG. Additionally, a control group of healthy patients without any anticoagulants, who had equivalent oral surgery, was added as a control group (named CG).

For each patient the following data was acquired from the digital patient file:

- Number of postoperative bleeding events (B0 = no bleeding event, B1 = one bleeding event, B2 = two bleeding events, B3 = three bleeding events, B4 = four bleeding events).
- Surgical intervention and postoperative bleeding events (tooth extraction, tooth extraction and osteotomy, osteotomies).
- Anticoagulation monitoring.
- Correlation of postoperative bleeding events and basic diseases..

Patients with incomplete documentation of the above listed information were not considered for the study. The primary outcome of the study was the frequency of postoperative bleeding in each group. Secondary outcomes were the type of surgery (tooth extraction with or without osteotomy), the number of extracted teeth and the INR. Ethical approval was obtained from the local medical faculty ethics committee (registration No.192_19Bc).

### Statistical analysis

Statistical analysis was performed using the statistical programming language R V3.6.1 (R Core Team (2019). R: A language and environment for statistical computing. R Foundation for Statistical Computing, Vienna, Austria). The non-parametric Kruskal–Wallis and Mann–Whitney U tests were used, as well as the Chi-square test, Fisher’s exact test and Cochran–Mantel–Haenszel test. The level of significance was *p* < 0.05 in all tests performed.

## Results

### General patient data

As a result of the data analysis, a total of 475 patients were included in the study, distributed to the three groups as follows: Bridging group (BG: n = 135), vitamin K antagonist group (VG: n = 170) and control group (CG: n = 170). The overall mean age was 71.76 years. Mean age in the groups was as follows: 79.67 years (BG), 78.76 years (VG) and 58.49 years (CG) with a statistical significance between CG and BG/VG (*p* < 0.001).

### Postoperative bleeding events following oral surgery

**Fig. 1:**
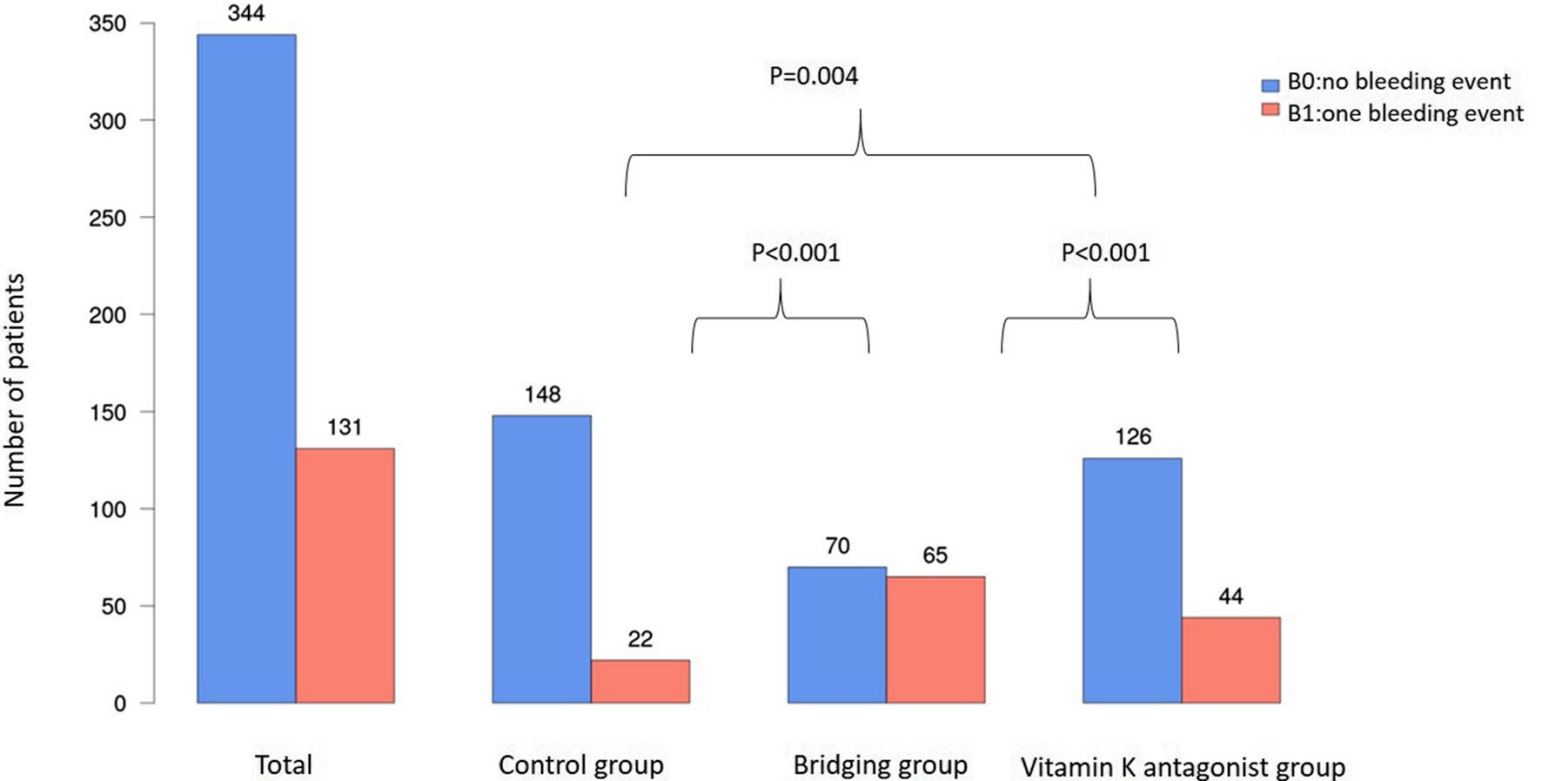
Showing the postoperative bleeding events within the different groups in relation to the interventions. B0 = no postoperative bleeding, B1 = postoperative bleeding event and the significances between groups (control vs. vitamin k antagonist *p* = 0.004; control vs. bridging group *p* < 0.001, bridging vs. vitamin k antagonist group *p* < 0.001). Control group with patients n = 170, bridging group n = 135, vitamin k antagonist group n = 170

Postoperative bleeding occurred in 22 out of 170 patients (12.9%) in the control group, in 44 out of 170 patients (25.9%) in the vitamin K antagonist group and in 65 out of 135 patients (48.1%) in the Bridging group. Comparing the groups statistically, significant differences were found for the control group versus the Bridging group (*p* < 0.001) and the control group versus the vitamin K antagonist group (*p* = 0.004). Furthermore, bleeding occurred significantly more often in the Bridging group than in the vitamin K antagonist group (*p* < 0.001), as shown in Fig. 1. The average number of postoperative bleeding events was 0.15 in the control group, 0.74 in the Bridging group and 0.29 in the vitamin K antagonist group, the number of postoperative bleeding events in the groups is presented in Table 1. There were significantly less bleeding events in the control group compared to the groups of anticoagulated patients (CG vs. BG: *p* < 0.001; CG vs. VG: *p* = 0.002). The comparison of the Bridging group and the vitamin K group revealed a higher number of bleeding events in the Bridging group (*p* < 0.001).

**Table 1:** Showing the number of postoperative bleeding events within the three groups. B0 = no postoperative bleeding, B1 = postoperative bleeding, B2,B3,B4 = two, three and four postoperative bleeding events. Control group with patients n = 170, bridging group n = 135, vitamin k antagonist group n = 170

B	Control group (n = 170)	Bridging group (n = 135)	Vitamin k-inhibitor group (n = 170)
0	148	70	126
1	20	40	38
2	1	17	0
3	1	6	0
4	0	2	0

### Number and techniques of tooth removal

**Fig. 2:**
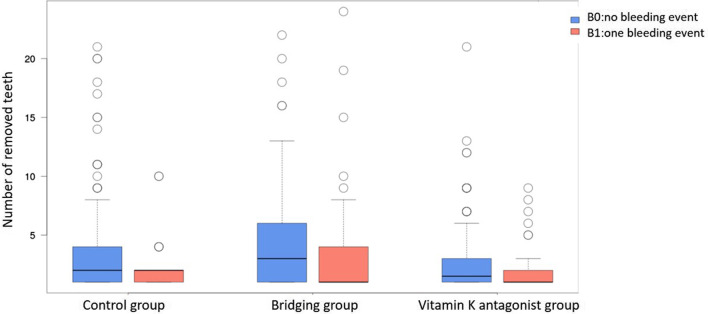
Showing the postoperative bleeding events within the different groups (control group, bridging group, vitamin k antagonist group) in relation to the number of extracted teeth. B0 = no postoperative bleeding, B1 = postoperative bleeding. With a significant correlation in the BG (*p* = 0.014) and no significance in VG (*p* = 0.298) and CG (*p* = 0.210)

**Fig. 3:**
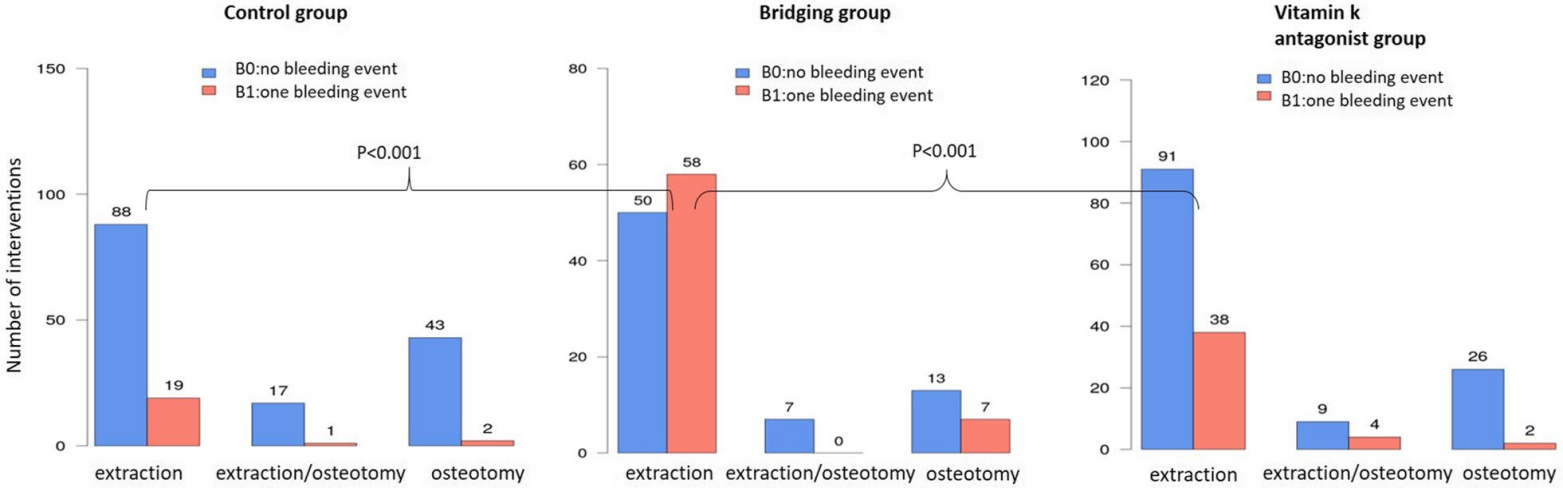
Showing the postoperative bleeding events within the groups (CG, BG and VG) in correlation to the surgical intervention (extraction; extraction and osteotomy; osteotomy). With no significances for osteotomy and extraction/osteotomy but within extraction in the BG to the VG and CG with *p* < 0.001

A total of 584 teeth were removed in the control group, 520 teeth in the Bridging group and 443 teeth in the vitamin K antagonist group. The procedures varied from single tooth extractions to row extractions of up to 24 teeth as a maximum. On average, 3.44 teeth were removed per procedure in the control group, 3.85 teeth in the Bridging group and 2.61 teeth in the vitamin K antagonist group. No significant difference was determined between the number of removed teeth and the number of postoperative bleeding events per procedure in the control group (*p* = 0.210) and in the vitamin K antagonist group (*p* = 0.298). The number of teeth had a significant impact on postoperative bleeding events in the bridging group (*p* = 0.014) as shown in Fig. 2. Teeth were removed by different surgical techniques, either by extraction or osteotomy or a combination of both. For the techniques osteotomy and combined extraction/osteotomy no significant differences were found related to bleeding events in the groups. For the technique tooth extraction there was significantly more postoperative bleeding found in the Bridging group compared to the control group (*p* < 0.001) and the vitamin K antagonist group (*p* < 0.001) as shown in Fig. 3.

### Anticoagulation monitoring

The Prothrombin time (PT) and International normalized ratio (INR) had been routinely determined before surgery and additionally when postoperative bleeding occurred. Bridging group patients without postoperative bleeding had an average INR of 1.4, whereas the average INR of those with bleeding was 1.45. In the vitamin K antagonist group the average INR was 2.1 for patients without bleeding and 2.7 for those with bleeding. The INR differed significantly between the groups of Bridging and vitamin K antagonist medication (*p* < 0.001), but it did not significantly differ between the groups of bleeding/non-bleeding patients (*p* > 0.05).

### Correlation of postoperative bleeding events and basic diseases

The basic diseases requiring anticoagulation therapy and the correlation with postoperative bleeding events within the two groups is illustrated in Table 2. Using odds ratios to indicate, whether the risk of postoperative bleeding event occurance is greater if the specific basic disease is present or not. With an odds ratio = 1, the risk is 0%, with an odds ratio > 1.0, the risk is increased, and with an odds ratio < 1.0, the risk is correspondingly decreased. No significance can be shown between the basic disease and postoperative bleeding events.

**Table 2:** Showing the *p* values > 0.05 (without two exceptions as marked) within the two groups in relation to the basic diseases and bleeding events after the Fisher’s exact test. With the Bonferroni correction, the significance level was calculated to *p* < 0.001 (0.05/42 = 0.001) without any signifincance between the groups and bleeding events in relation to the basic disease

	Bridging	Odds ratio	*p* value	Marcumar	Odds ratio	*p* value
	n/%			n/%		
Arrythmia	78 (57.8%)	1.52	0.3	96 (56.5%)	2.31	0.03
Coronary heart disease	22 (16.3%)	1.36	0.64	23 (13.5%)	1.21	0.8
Embolism	9 (6.7%)	0.85	1	10 (5.9%)	0.7	1
Chronic heart failure	15 (11.1%)	1.26	0.79	17 (10%)	1.65	0.38
Thromboses (> 6 months)	10 (7.4%)	0.11	0.02	13 (7.6%)	0.5	0.52
Apoplexy	17 (12.6%)	1.25	0.8	19 (11.2%)	0.74	0.78
Artificial heart valve	21 (15.6%)	1.53	0.48	32 (18.8%)	0.61	0.37
Cardiac pacemaker	18 (13.3%)	1.41	0.61	25 (14.7%)	1.14	0.81
Peripheral arterial disease	6 (4.4%)	0.52	0.68	7 (4.1%)	2.23	0.38
Bypass	6 (4.4%)	1.08	1	13 (7.6%)	0	0.02
Cardiomyopathy	5 (3.7%)	0.71	1	4 (2.4%)	0	0.57
Myocardial infarction	7 (5.2%)	0.8	1	11 (6.5%)	1.08	1
Heart transplant	0 (0.0%)	n.a	n.a	3 (1.8%)	0	0.57
Stent implant	6 (4.4%)	1.08	1	7 (4.1%)	2.23	0.38
Valvular heart disease	3 (2.2%)	2.19	0.61	2 (1.2%)	2.91	0.45

## Discussion

Anticoagulated patients continue to pose a challenge in everyday clinical practice [18]. This is particularly true for surgical procedures, including oral surgery. On the one hand, discontinuation or Bridging of anticoagulation can lead to thromboembolic events with a potentially lethal outcome [4, 19]. On the other hand, intra- and postoperative bleeding can be burdensome for the patient and may complicate surgery and wound healing. Nevertheless it can be controlled sufficiently by local hemostatic measures in the majority of cases [20]. As a result, the question whether to perform perioperative Bridging or to continue vitamin K antagonist medication in oral surgery, is becoming an increasingly contentious issue. There seems to exist a vague consensus pro Bridging when it comes to major surgical procedures such as extensive oncological or reconstructive operations, but for small to moderate surgical procedures, opinions and study results about the perioperative anticoagulation management differ widely. Clemm et al. investigated bleeding complications of anticoagulated patients in dental implant surgery (implant insertion and augmentative procedures). Comparing different anticoagulative schemes, they found a bleeding risk of 12.5% in a Bridging group (low-molecular-weight heparin), 6.7% in the vitamin K antagonist group, 1.4% in a platelet aggregation inhibitor group and 0.6% in a control group [21]. In another study by Bajkin et al. no significant differences in terms of postoperative bleeding following dental surgery were found between the Bridging and Non-Bridging groups of a 214 patients cohort. In their 2015 systematic review, Kämmerer et al. found a strong evidence, that patients with vitamin K antagonist medication undergoing minor oral surgery should not discontinue their medication in order to prevent thromboembolic complications [2]. This corresponds with the findings of our study. The probability for the occurrence of postoperative bleeding as well as the frequency of bleeding events were significantly higher in the Bridging group compared to the vitamin K antagonist group. As expected, the INR was significantly lower in the vitamin K antagonist group than in the Bridging group, but surprisingly there were no significant differences found within the groups comparing bleeding and non-bleeding patients. Other studies showed similar findings regarding the INR by not finding a correlation of bleeding events with the INR [22]. In a study by Schmitt et al. in 2019, the INR (mean value in the bridging group, 1.67; mean value in vitamin-k-inhibitor group, 1.8) had no significant association with postoperative bleeding events. The incidence of bleeding events in the vitamin K antagonist group was 11.3%, which is quite similar to our result. In contrast, the bridging group, with an incidence of 0%, did not record a single event. However, the bridging group consisted of only 6 patients, and the vitamin-k-inhibitor group included 80 patients [23].

Postoperative bleeding event, were also recorded in correlation with the surgical intervention (single tooth extraction, serial tooth extraction or osteotomy). Single tooth extraction within the vitamin K antagonist group resulted in a rate of postoperative bleeding events of 10.5%, a rate of serial extraction of 16.7% and a rate of osteotomy of 10%. In the control group with 603 procedures, they found 0% postoperative bleeding events in single tooth and serial extractions and 1.3% in osteotomies. These results correspond to those of another study where 214 patients, who underwent tooth extraction of one to five teeth per procedure without a significant correlation [24]. In our patient population, the occurrence of postoperative bleeding significantly correlated with the number of teeth removed within the bridging group and in terms of the surgical intervention extraction but not within osteotomy or within the other groups (VG and CG). This does not correspond with the findings of another author. Bleeding did not correlate with the extension of the surgical procedure [2]. One reason for the increased postoperative bleeding in correlation with the number of teeth removed in the BG within this study may be that external patients were also included. Currently bridging of vitamin k is still common practice beyond the university hospitals for tooth removal. Therefore, it was not possible to differentiate between the other influencing factors (such as wound management or invasiveness during tooth extraction) that might be crucial in terms of bleeding, especially in the BG but also CG and VG. As these data could not be collected due to the retrospective design of the study.

In a review, Wahl et al. examined more than 2775 patients with dental procedures under bridging with heparin conditions. Postoperative bleeding occurred in 161 patients (6%), which needed intervention in four patients (0.14%) with more than local hemostatic measures [4]. Additionally, other studies showed that local hemostatic measures were sufficient for hemostasis in most dental interventions of anticoagulated patients and that possible bleeding complications in anticoagulant patients undergoing dental surgery should be weighed against possible embolism complications before anticoagulation is bypassed [10, 25–27]. In our patients, we observed, that only in the Bridging group local hemostatic measures had to be escalated in the case of multiple bleeding events. The observations in our patient population correlate with the findings of other studies and lead to the conclusion that patients do not benefit from Bridging in dental surgery [28]. It was not possible to draw a line between the measures without too much bias within this patient population. This was because external patients with postoperative bleeding events were also included in the study. Thus, to a certain extent, no action cascade of the hemostatic measures could be carried out. These range from Tranexamic acid (Cyklokapron®, Pfizer Pharma GmbH, Berlin, Germany) with a bite swab and local compression, inserting hemostatic fillers (i.e. Oxycellulose, Tabotamp® Johnson & Johnson Medical GmbH, Norderstedt, Germany; Collagen, Lyostypt® B. Braun Melsungen AG, Melsungen, Germany; Porcine gelatin, Gelastypt® Sanofi-Aventis Germany GmbH, Frankfurt am Main, Germany), bipolar electrocoagulation or bandage plate (acrylic splints) and local tight wound closure to stop postoperative bleeding.

However, also the continuation of vitamin K antagonists still poses a challenge. This is because even in this case, inconsiderable secondary bleeding may occur, although it can be easily treated by local hemostatic measures [2, 4, 5, 22]. Most medical specialists recommend adjusting or reducing the INR value without permanently leaving the therapeutic area [4]. The risk of a lethal thromboembolic event, which is 0.2% in the literature and should not be disregarded [4]. The current guidelines of the American Heart Association and many other professional societies recommend adherence to two important key points. Oral anticoagulation should not be suspended during procedures with a low risk of bleeding. Patients at high thromboembolic risk without a high risk of bleeding should be bridged, while those at correspondingly low thromboembolic risk should not. In patients who are in the acute phase (3–6 months) after a thromboembolic event, all surgical procedures should be postponed if possible [29]. Accordingly, the professional societies classify dental surgical procedures as a low risk of bleeding. In this study, we were not able to establish a correlation between a postoperative bleeding event and the basic disease, which needs anticoagulation therapy within this study. Neither in the bridging nor in the non-paused vitamin K group.

There are shortcomings of this study that need to be discussed. First, the retrospective study design led to discrepancies between the groups in terms of group size and composition. The extent and type of the surgical procedure varied between the groups and since operations were performed by different surgeons, the surgical techniques varied to a certain extent. Furthermore, a possible confounding factor of different platelet counts within the groups could not be included in the evaluation.

## Conclusions

Within the limitations of the current study, it can be concluded that postoperative bleeding events occur significantly more frequently in bridged patients than in patients with un-paused vitamin K antagonist medication. It therefore appears reasonable to continue vitamin K antagonist medication perioperatively for the investigated class of small-to-medium sized oral surgery cases. A close interdisciplinary collaboration between oral surgeons and other medicine specialists is essential to minimize perioperative risks for the patients.

## Data Availability

The datasets used and/or analyzed during the current study are available from the corresponding author on reasonable request.

## Abbreviations

BG: Bridging group
VG: Vitamin K antagonist group
CG: Control group
PT: Prothrombin time
INR: International normalized ratio
